# Linear IgA Bullous Dermatosis in an Elderly Patient Following Meloxicam and Candesartan Use: A Case Report

**DOI:** 10.7759/cureus.99054

**Published:** 2025-12-12

**Authors:** Shatha Albyali, Nora Alhedaithi, Renad Alqahtani, Rinad Alenazi, Faisal Alsharif, Ahmed Alhumidi, Saleh Alkhamees

**Affiliations:** 1 Department of Dermatology, College of Medicine, Alfaisal University, Riyadh, SAU; 2 College of Medicine, Princess Nourah Bint Abdulrahman University, Riyadh, SAU; 3 Department of Dermatology, Security Forces Hospital, Riyadh, SAU; 4 Department of Pathology, College of Medicine, King Saud University, Riyadh, SAU

**Keywords:** autoimmune skin disease, candesartan, drug-induced blistering disorders, linear iga bullous dermatosis, meloxicam

## Abstract

Linear immunoglobulin A (IgA) bullous dermatosis (LABD) is a rare autoimmune blistering disorder characterized by subepidermal blisters and linear IgA deposition along the dermo-epidermal junction (DEJ). We report the case of a 61-year-old woman who developed multiple tense vesicles and bullae on an erythematous base affecting both upper and lower extremities after initiating meloxicam for postoperative analgesia following knee arthroplasty. A punch biopsy showed subepidermal blistering, while direct immunofluorescence demonstrated linear IgA deposition along the basement membrane zone, confirming LABD. After discontinuation of the suspected culprit drug and adjustment of her antihypertensive therapy, she was treated with oral dapsone 1 mg/kg (50 mg) daily for two weeks, resulting in clinical improvement. This case underscores the importance of thorough drug history taking and early recognition of drug-induced LABD, particularly in patients on multiple medications, where identifying the true offending agent may be challenging.

## Introduction

Linear immunoglobulin A (IgA) bullous dermatosis (LABD) is a rare autoimmune vesiculobullous disorder. It is characterized by the presence of IgA autoantibodies targeting antigens within the basement membrane zone of the skin and/or mucosa [1]. Although the majority of LABD cases are considered idiopathic, a distinct subset has been temporally and clinically associated with the administration of specific pharmacological agents. Among these, antithrombotic, antiplatelet, and anticancer drugs have been implicated in the development of bullous lesions. However, the precise causal relationship between specific medications and LABD remains incompletely understood, and considerable interindividual variability in response has been observed [2,3]. The diagnosis of linear LABD is confirmed by demonstrating linear IgA deposition along the basement membrane zone on direct immunofluorescence (DIF), together with subepidermal blistering [4].

The incidence of LABD is estimated at approximately one case per one million person-years. The clinical onset of LABD is highly heterogeneous and exhibits considerable polymorphism. Characteristic manifestations include polycyclic arrangements of bullae with central crusting, commonly referred to as a “cluster of jewels” or “string of pearls,” which are particularly prominent in spontaneous childhood forms [5,6]. Nevertheless, the clinical phenotype may be markedly variable, and rare presentations resembling toxic epidermal necrolysis (TEN) have also been documented [7,8].

Vancomycin has been consistently reported as the most frequent causative agent, and several other antibiotics, non-steroidal anti-inflammatory drugs (NSAIDs), and antihypertensive agents have been implicated. However, there is limited published evidence documenting LABD triggered specifically by meloxicam (an NSAID) and candesartan (an angiotensin II receptor blocker (ARB)), particularly in elderly patients [9,10]. The co-administration of these drugs as a potential trigger for LABD has not been systematically reported, leaving a gap in understanding the spectrum of drug-induced LABD and its risk factors in older adults.

The aim of this study is to report a rare case of drug-induced LABD in an elderly patient following the use of meloxicam and candesartan and to highlight the clinical, histopathological, and immunofluorescence features that support diagnosis. Additionally, the study aims to emphasize the importance of comprehensive drug history assessment and early recognition of drug-induced LABD in patients receiving multiple medications.

## Case presentation

A 61-year-old Saudi woman with a history of hypothyroidism (on levothyroxine), hypertension (on candesartan cilexetil), pityriasis lichenoides chronica, psoriasis, and urge urinary incontinence (on mirabegron) presented with a four-day history of multiple tense bullae over the upper and lower limbs. The lesions progressively spread to involve the back and trunk and were associated with severe pruritus, particularly at night, which significantly impaired her daily activities.

**Figure 1:**
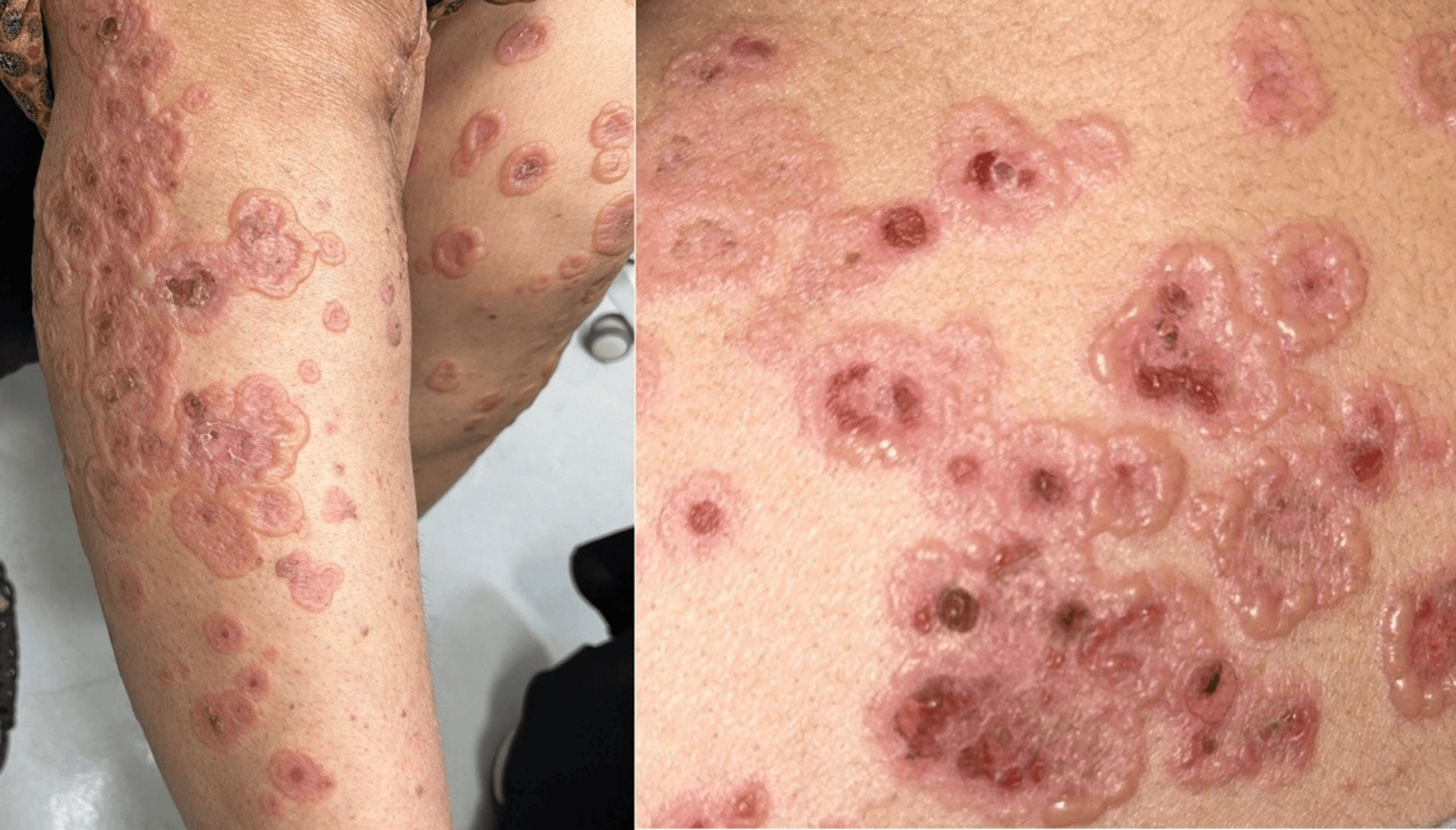
Multiple single and grouped, round, fluid-filled lesions consistent with tense bullae, exhibiting the characteristic “crown of jewels” sign

Her surgical history was notable for recent knee arthroplasty, after which she was prescribed meloxicam for postoperative analgesia, which she had continued. Examination revealed multiple, round, fluid-filled, tense bullae-both single and grouped-distributed over the upper and lower limbs, displaying the classic “crown of jewels” sign (Figure 1). The oral mucosa was affected, while the genital mucosa was spared. The overall body surface area involvement exceeded 10%.

Differential diagnoses included bullous pemphigoid and LABD. Laboratory investigations were largely unremarkable except for anemia (Hb 10.1 g/dL (reference range: 12-15.5)) and leukocytosis (13.1 ×10³/µL (reference range: 4-11)). Glucose-6-phosphate dehydrogenase activity, liver, and renal function were normal.

**Figure 2:**
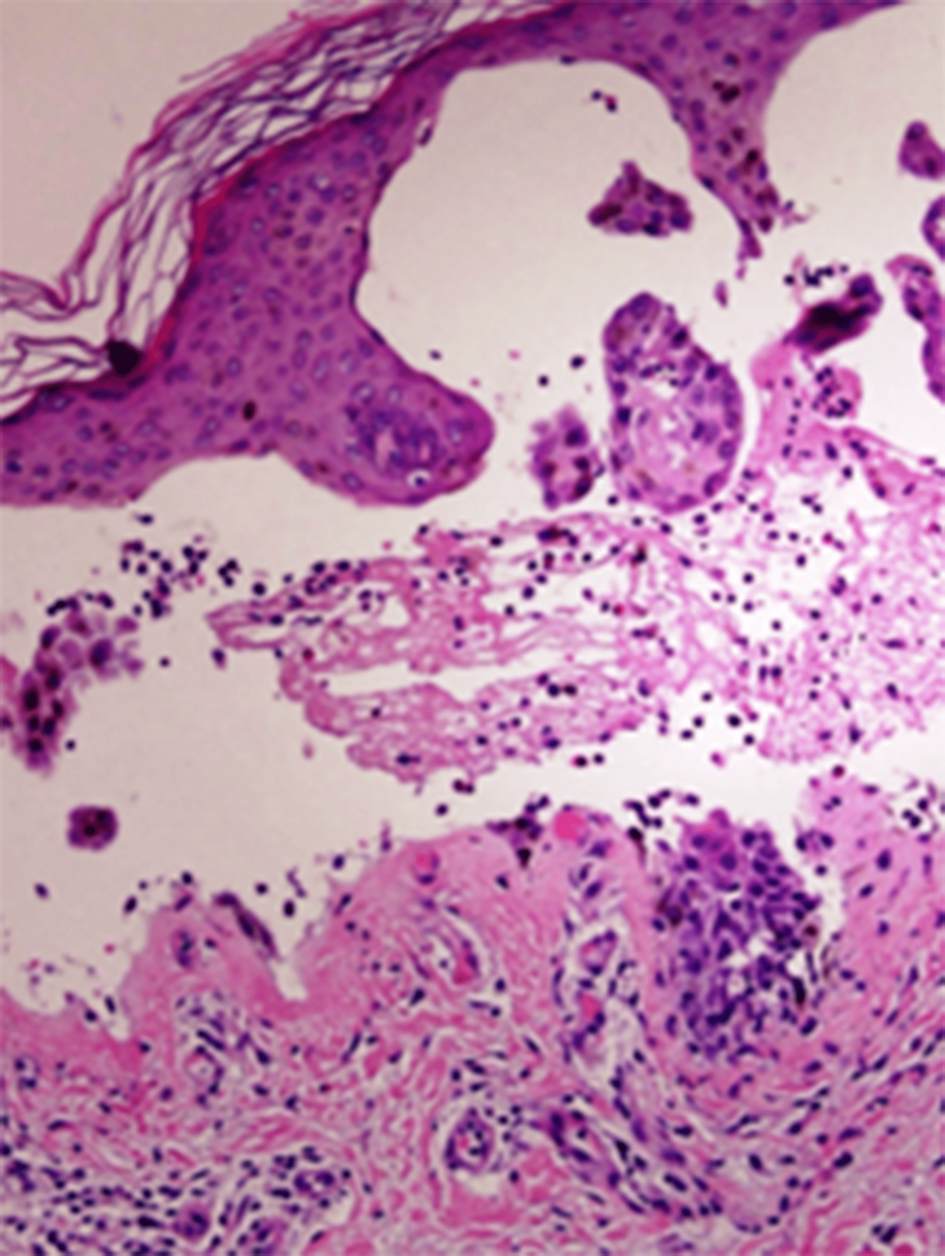
Microscopic examination of the skin punch biopsy showing a subepidermal blister with a predominantly neutrophilic infiltrate (hematoxylin and eosin stain, magnification: ×400)

**Figure 3:**
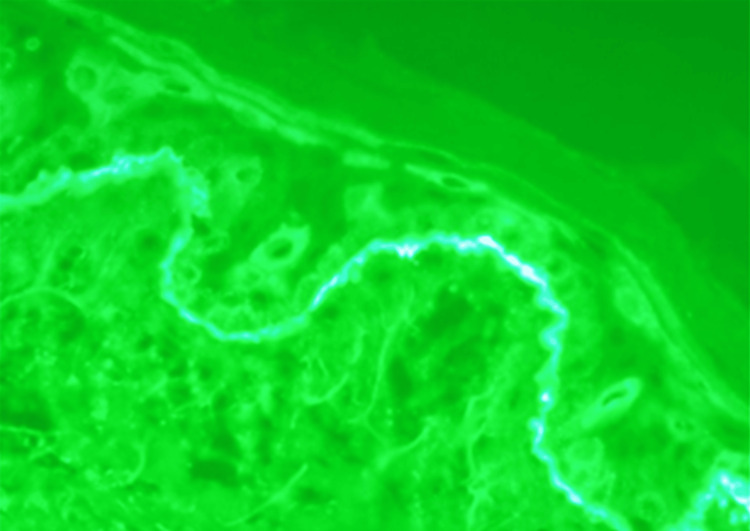
Direct immunofluorescence (DIF) of perilesional skin demonstrating a bright, continuous, linear deposition of IgA along the basement membrane zone, consistent with linear IgA bullous dermatosis (Magnification: ×400)

A punch biopsy showed a subepidermal blister containing fibrin and numerous neutrophils, with occasional eosinophils (Figure 2). Direct immunofluorescence (DIF) performed on perilesional skin revealed bright, continuous, linear IgA deposition along the basement membrane zone, without significant IgG or C3 deposition (Figure 3). These findings confirmed LABD.

Meloxicam was discontinued, and her antihypertensive regimen was modified. She started on oral dapsone 1 mg/kg (50 mg) daily for two weeks, leading to significant clinical improvement.

## Discussion

Although LABD is an uncommon dermatologic entity, its clinical relevance is amplified by its association with a broad range of medications. Clinically and pathologically, drug-induced cases may exhibit considerable variability, which can complicate timely recognition and diagnosis. In this case, a 61-year-old woman developed tense vesicles and bullae on her extremities four days after initiating meloxicam, while on candesartan. Histopathology and direct immunofluorescence confirmed LABD. Withdrawal of the suspected drugs and initiation of oral dapsone led to rapid clinical improvement. This case emphasizes the importance of prompt recognition of drug-induced LABD, careful review of all current medications in elderly patients, and early discontinuation of potential offending agents to prevent disease progression and minimize morbidity. This case is notable for the concurrent involvement of two infrequently reported agents, meloxicam and candesartan, as potential triggers of LABD. Furthermore, the presence of preexisting autoimmune dermatologic conditions further complicated the diagnostic evaluation.

The occurrence of LABD in association with meloxicam or candesartan individually has been documented, albeit rarely [9]. However, the simultaneous implication of both agents as potential triggers in a single patient is exceedingly uncommon, with no previously reported cases in the literature to our knowledge. Lammer et al. identified 76 drugs associated with LABD, including NSAIDs and antihypertensives [3]. Pharmacovigilance data have identified 17 reported cases of meloxicam-associated LABD [11], and candesartan has likewise been implicated in isolated case reports [12]. Additionally, a previous report described an LABD presentation overlapping with drug reaction with eosinophilia and systemic symptoms (DRESS) in a patient receiving meloxicam in combination with amlodipine [13]. These findings highlighted that the concurrent use of meloxicam and candesartan as potential triggers of LABD, though exceedingly rare, highlights the importance of thorough medication review in patients with new-onset blistering lesions. Early recognition, prompt withdrawal of offending agents, and careful monitoring in elderly patients with comorbidities are essential to prevent disease progression and optimize outcomes.

In the present case, the patient developed cutaneous lesions four days after initiating the suspected medications, supporting a temporal pattern consistent with drug-induced LABD. This aligns with existing literature indicating that most causative medications are typically started within four weeks prior to disease onset [14]. In contrast, previous studies have shown that patients with spontaneous (non-drug-induced) LABD often report medication use for more than 12 months before lesion development, making long-term therapies less likely to be implicated as triggers [11]. Mucosal involvement is observed in up to 80% of adult patients with idiopathic LABD, whereas drug-induced cases typically spare mucosal surfaces [15]. In the present case, the oral mucosa was involved while the genital mucosa remained unaffected, and the overall body surface area involvement exceeded 10%, underscoring the importance of considering a drug-induced etiology when mucosal involvement is limited, which may influence both diagnosis and management strategies.

Our patient improved rapidly after discontinuing meloxicam, adjusting the antihypertensive therapy, and initiating short-term oral dapsone. This highlighted the importance of withdrawing the offending drug and using targeted therapy to control inflammation in drug-induced LABD. Parallel management of drug-induced LABD typically involves discontinuation of the offending agent, which is often sufficient to induce clinical improvement. Dapsone is widely recognized as first-line therapy and has demonstrated high efficacy in controlling inflammation and promoting lesion resolution [3]. In more severe or atypical presentations, systemic corticosteroids may be added to accelerate disease control. Careful monitoring during therapy, particularly in elderly patients, is essential to detect potential adverse effects and ensure safe and effective treatment [16].

The patient’s preexisting autoimmune dermatoses, including psoriasis and pityriasis lichenoides chronica, may have increased her susceptibility to drug-induced LABD. LABD has been reported to coexist with other autoimmune conditions, suggesting a shared predisposition related to immune dysregulation [3,17]. This emphasizes the need for careful monitoring and heightened vigilance when initiating potentially triggering medications in patients with underlying autoimmune diseases. Recognizing this predisposition can guide clinicians in early diagnosis and tailored management strategies to minimize complications.

This report is limited by its single-patient design and the inability to definitively establish causality between LABD and concurrent meloxicam and candesartan use. Larger case series and pharmacovigilance studies are needed to better define drug triggers, identify predisposing factors, and guide management. Additionally, long-term follow-up and mechanistic studies could provide insights into the immunopathogenesis of drug-induced LABD, especially in patients with underlying autoimmune diseases.

## Conclusions

This case presents a rare instance of drug-induced LABD linked to meloxicam and candesartan in an elderly patient with autoimmune comorbidities. The rapid onset of painful bullae after medication use, along with supportive histopathology and immunofluorescence, emphasizes the need to consider recent drug exposures in new blistering diseases.

As older adults frequently take multiple medications and may be more prone to immune-mediated reactions, clinicians should remain aware of even uncommon drug triggers. Continued pharmacovigilance and research are essential to better understand, prevent, and manage drug-induced LABD.
